# Management Recommendations for Merkel Cell Carcinoma—A Danish Perspective

**DOI:** 10.3390/cancers12030554

**Published:** 2020-02-28

**Authors:** Simon Naseri, Torben Steiniche, Morten Ladekarl, Marie Louise Bønnelykke-Behrndtz, Lisbet R. Hölmich, Seppo W. Langer, Alessandro Venzo, Elizaveta Tabaksblat, Siri Klausen, Mathilde Skaarup Larsen, Niels Junker, Annette H. Chakera

**Affiliations:** 1 Department of Pathology, Aarhus University Hospital, 8200 Aarhus, Denmark; 2 Department of Oncology, Clinical Cancer Research Center, Aalborg University Hospital, 9000 Aalborg, Denmark; 3 Department of Plastic and Reconstructive Surgery, Aarhus University Hospital, 8200 Aarhus, Denmark; 4 Department of Plastic Surgery, Herlev & Gentofte Hospital, Department of Clinical Medicine, Copenhagen University, 2730 Herlev, Denmark; 5 Department of Oncology, Copenhagen University Hospital, Rigshospitalet, 2100 Copenhagen, Denmark; seppo.langer@regionh.dk; 6 Department of Plastic Surgery and Burns Treatment, Rigshospitalet, Copenhagen University Hospital, 2100 Copenhagen, Denmark; 7 Department of Oncology, Aarhus University Hospital, 8200 Aarhus, Denmark; 8 Department of Pathology, Herlev Hospital, 2730 Herlev, Denmark; 9 Department of Oncology, Herlev Hospital, 2730 Herlev, Denmark

**Keywords:** Merkel cell carcinoma, diagnosis, treatment, review, guideline

## Abstract

Merkel cell carcinoma (MCC) is a rare malignant neuroendocrine carcinoma of the skin with a poor prognosis and an apparent increase in incidence. Due to its rarity, evidence-based guidelines are limited, and there is a lack of awareness among clinicians. This review constitutes the consensus management recommendations developed by the Danish MCC expert group and is based on a systematic literature search. Patients with localized disease are recommended surgical excision and adjuvant radiotherapy to the primary site; however, this may be omitted in patients with MCC with low risk features. Patients with regional lymph node involvement are recommended complete lymph node removal and adjuvant radiotherapy in case of extracapsular disease. Metastatic disease was traditionally treated with chemotherapy, however, recent clinical trials with immune therapy have been promising. Immune checkpoint inhibitors targeting the programmed cell death protein 1(PD-1)/programmed death-ligand 1(PD-L1) axis should therefore be strongly considered as first-line treatment for fit patients. A 5-year follow-up period is recommended involving clinical exam every 3 months for 2 years and every 6 months for the following 3 years and PET-CT one to two times a year or if clinically indicated. These national recommendations are intended to offer uniform patient treatment and hopefully improve prognosis.

## 1. Introduction

Merkel cell carcinoma (MCC) is a rare and highly aggressive neuroendocrine malignancy of the skin. Research in MCC has recently gained traction due to successful clinical trials with immune checkpoint inhibitors and the discovery of the Merkel cell polyoma virus (MCPyV) [1,2]. The incidence in Denmark has increased 5.4-fold from 0.06 cases/100,000 in 1986 to 0.31 cases/100,000 in 2002 [3]. The median age of patients at diagnosis is 77 years, with the majority being male (62%) [4]. MCC is primarily caused by UV-radiation (24%) and/or the presence of MCPyV (76%) [1,5,6]. Immunosuppression also plays a role and increases the risk of MCC, as seen in individuals with chronic lymphatic leukemia (30-fold), the human immunodeficiency virus/acquired immunodeficiency syndrome (HIV/AIDS, 13-fold) and organ transplants recipients (10-fold) [7]. Currently, the 5-year overall survival of MCC is approximately 40%, making it more deadly than melanoma [8]. The current review constitutes the national Merkel cell carcinoma management recommendations developed by the Danish MCC expert group.

## 2. Diagnosis

### 2.1. Clinical Features

**Figure 1:**
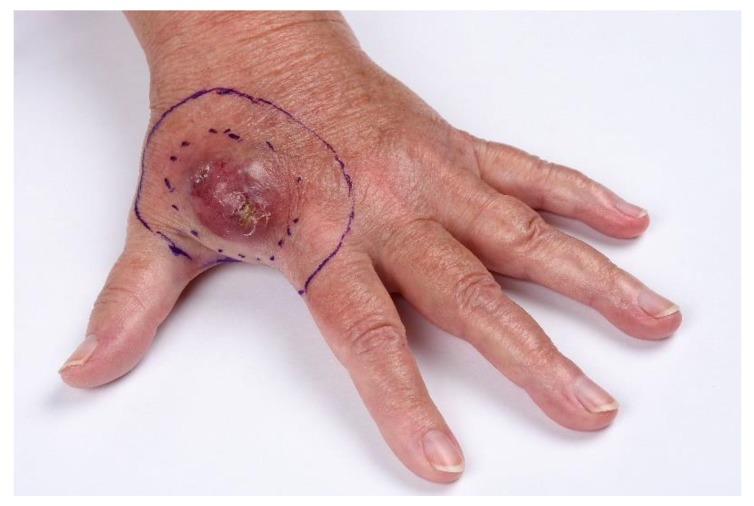
A red, nodular primary MCC on the left hand.

MCC usually presents as a nonspecific, firm, rapidly growing, painless red, purple or pink exophytic cutaneous nodule (Figure 1) [9]. The tumor is most often located in the head and neck area (43%), followed by the extremities (39%) [8]. As the presentation is often nonspecific and clinical diagnosis is only correct in 1% of cases, the AEIOU acronym has been developed to increase diagnostic accuracy by defining clinical features of MCC: Asymptomatic/lack of tenderness, Expanding rapidly, Immune suppression, Older than 50 years, and Ultraviolet-exposed site on a person with fair skin. Approximately 89% of patients have ≥3 of these features. Common benign misdiagnoses include cyst/acneiform lesion, lipoma, fibroma, vascular lesion or insect bite [9].

### 2.2. Pathology

Histology: MCC is often located in the dermis extending into the subcutaneous tissue. The cells are small, uniform and basaloid with granulated chromatin, scant cytoplasm and high mitotic rates [10]. Necrosis, increased vascularization, immune cell infiltration, solar elastosis and collision tumors (squamous cell carcinoma, basal cell carcinoma, actinic keratosis, Bowen disease) are common features [10]. Having the appearance of a small round blue cell tumor, MCC has many differential diagnoses, the most important being basal and squamous cell carcinomas, metastatic small cell carcinomas from other sites, melanoma, non-Hodgkin lymphomas and anaplastic adnexal carcinomas [11].

Immunohistochemistry (Table 1): MCC most commonly expresses epithelial markers, more specifically CK20, with a paranuclear dot-like staining. It shows neuroendocrine features with expression of neuroendocrine markers such as CD56 (88.2% positivity), chromogranin A (84.1% positivity) and synaptophysin (92% positivity) [12].

MCC is negative for leukocyte common antigen (CD45, in contrast to lymphoma), S-100 (in contrast to melanoma), CK7 and TTF-1 (in contrast to small cell lung cancer) [12,13].

MCC diagnosis is based on histological features combined with CK20 expression and TTF-1 negativity. A subgroup of MCC does not stain accordingly, as 12.6% of MCC are CK20 negative, while 7% express TTF-1 [12]. CM2B4, which targets the MCV-large T antigen, may also be useful in diagnostics of virus-positive MCC (sensitivity 88.2%; specificity 94.3%) [6].

Proposed immunohistochemical panel: CK(AE1/AE3), CK20, CK7, TTF-1, CD45, S-100, CD-99 and neuroendocrine markers, such as synaptophysin, chromogranin A and CD56. In case of a nonconclusive result of the immunohistochemical panel, it may be necessary to do supplementary staining according to differential diagnosis.

Sentinel node (SN) protocol: A formalin-fixed SN will be split through the hilum and optionally further cut into parallel slices. The paraffin-embedded tissue slices will be cut in six serial steps with 50 μm intervals with a HE and CK(AE1/AE3) stained section at each level [14]. Isolated tumor cells within a lymphatic channel in the parenchyma of a lymph node, or its capsule, are classified as a metastasis.

Pathology report content should include:

Histopathological parameters: Macroscopic tumor diameter (microscopic in case of no clinical estimation, horizontal, in mm); Margin status (distance from lateral and deep resection margins, macroscopic and/or microscopic estimation, in mm); Tumor thickness (from stratum granulare, or highest tumor cell in case of ulceration, to deepest tumor cell, in mm with no decimals); Ulceration (total loss of epidermis with vital reaction, present/absent); Lymphovascular invasion (LVI) and perineural invasion (present/absent); Level of invasion (extracutaneous extension to muscle/fascia/cartilage/bone); Collision tumors (description if present).

Immunohistochemical analyses: Ki67 (% of tumor cells, estimate in 10% intervals if image counting tools are not available (more reproducible than hotspot counting)); Tumor infiltrating lymphocytes (number of CD8 positive lymphocytes per HPF); Viral status (MCPyV is positive when >1% of tumor cells stain independently of intensity when using the immunohistochemical clone CM2B4).

Sentinel lymph node status: Maximum diameter of the metastasis is reported, as well as number of involved/removed lymph nodes and extracapsular extension (presence of nodal metastasis extending through the lymph node capsule and into adjacent tissue) [15]; In-transit metastasis (present/absent): A discontinuous tumor distinct from the primary lesion and located between the primary lesion and the draining regional lymph nodes or distal to the primary lesion (Figure S1).

## 3. Work-Up & Staging

### 3.1. Work-Up

**Figure 2:**
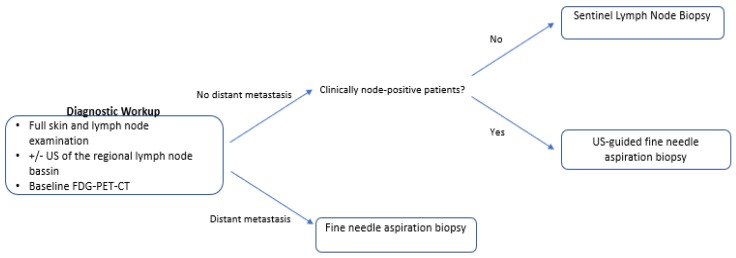
Diagnostic workup for MCC patients.

Patients should undergo full clinical skin and lymph node investigation with/without ultrasound (US) of the regional lymph node basin (Figure 2). Baseline F18-FDG whole-body positron emission tomography-computed tomography (PET-CT) scan may exclude differential diagnosis (mainly small cell lung cancer), lead to upstaging in 16% of patients and change management in up to 37% (Table 2) [16,17]. Until more data are produced, baseline PET-CT may be considered for all MCC patients.

Patients without clinically involved lymph nodes are recommended sentinel lymph node biopsy (SLNB), as 24–32% of these patients harbor clinically occult, microscopic regional metastasis [18,19,20]. Risk increases with tumor diameter, but patients with tumors down to 0.5 cm have been shown to be sentinel lymph node (SLN) positive in 14% [21]. SLNB should be carried out at the same time as wide excision to avoid inaccuracy and minimize the risk of false-negative results if performed after wide excision (15–17%) [18,22]. Patients with clinically involved lymph nodes should be offered US-guided fine needle aspiration biopsy (FNAB) to confirm the diagnosis.

### 3.2. Staging

The current staging is based on the eighth edition of the tumor, node, metastasis (TNM) staging system, recommend by the Union for International Cancer Control (UICC) and the American Joint Committee on Cancer (Figure S1). The expected 5-year overall survival (OS) for each stage is illustrated in Table 3 [8].

The eighth edition of the TNM staging system is based on 9387 MCC cases with follow-up and staging data from 1998 to 2012 [8]. Staging can be clinical or pathological; the latter is more precise. Patients with local disease and negative SLNs have better prognosis (76% at 5 years) than those with clinically negative nodes (59% at 5 years) as the latter may harbor occult metastases that are not detected at time of diagnosis if SLNB is not performed [23]. Patients with nodal disease and unknown primary tumor are now given similar stage as patients with any tumor and microscopic nodal involvement (pathological stage IIIA), as patients with clinically detected nodal disease and unknown primary tumor show improved prognosis over cases with concurrent known primary tumor (OS 42% vs. 27%, respectively) [8].

## 4. Treatment

Due to the rarity of MCC, prospective clinical trials are rarely conducted. Therefore, current treatment recommendations are mostly based on retrospective studies with few patients (Figure S2).

## 5. Management of the Primary Tumor

### 5.1. Surgery

Traditionally, the recommended excisional margin for primary tumors has been 2–3 cm [24,25]. Recent studies comparing different excision margins show that patients (*n* = 47) treated with 1, 2 or 3 cm margins did not have a statistically significant difference in disease-free survival and OS [26]. Similarly, the largest single-institution study to date (*n* = 240) did not demonstrate a significant difference in local recurrence or disease-specific survival between patients treated with 1, 1.1–1.9 or >2 cm excisions [27]. Surgery-only (*n* = 104) with an excisional width of 1–2 cm to the tumor bed (tumor diameter < 2 cm) has demonstrated local recurrence rates down to 1.9% [19]. However, these studies were not randomized clinical trials so confounding by indication may be prevalent; larger excision margins may have been used for larger tumors. Regular randomized trials testing different resection margins are warranted but difficult to complete due to the small number of patients. A positive surgical margin is associated with reduced OS and should lead to re-excision [28,29]. Based on the above studies, an excisional margin of 1–2 cm is recommended.

### 5.2. Adjuvant Radiotherapy

Primary tumor: Radiotherapy (RT) is recommended following surgical excision [30]. In 4843 MCC cases, the largest cohort to date, it was shown that localized MCC (stage I and II) treated with primary surgery and adjuvant RT was associated with improved OS, compared to surgery alone (stage I: HR = 0.71, 95% CI = 0.64 to 0.80, *p* < 0.001; stage II: HR 0.77, 95 % CI = 0.66 to 0.89, *p* < 0.001) [28].

Recommended dose is 50–60 Gy at 2 Gy/d, 5 fractions per week (F/W) [31,32,33]. Adjuvant radiotherapy (RT) to the primary site has been shown to improve local control, and data from three pooled prospective trials, which included 88 high-risk MCC patients, showed that pre-radiation margin status (positive/negative) did not have an impact on time to loco-regional failure in patients receiving adjuvant RT [34]. As most MCCs are located in the head-and-neck area, a wide surgical margin is not always feasible and should not be pursued at all costs, but respect functionality and cosmesis, especially as adjuvant RT leads to a high degree of local control. Administration of RT should be carried out within 3 weeks after surgery to minimize disease progression prior to RT [35].

Adjuvant RT may be left out in patients with low-risk characteristics in their primary tumors (Figure S3). These include small primary tumors (≤1 cm diameter), negative margin status, no LVI, negative SLNB and no chronic immunosuppression (i.e., lymphoma/leukemia) [18,19,36]. In a small retrospective study on patients with low-risk head-and-neck primary tumors, adjuvant RT was associated with increased local control without a survival benefit [37]. Since all recurrences were salvaged by radiotherapy, adjuvant RT should not routinely be recommended for this patient subgroup but discussed per case.

Regional lymph nodes: Prophylactic regional RT is not recommended in SLNB-negative patients, as this has not shown to reduce the regional recurrence rate [38].

### 5.3. Definitive Radiotherapy—Nonresectable Disease

Definitive RT increases disease control but should be reserved for patients who are not candidates for complete, gross resection or refuse surgical intervention. A systematic review including 23 studies found that definitive RT to 136 primary tumor sites resulted in local recurrence rates of 7.6% with a median follow-up time of 24 months. Definitive RT was more effective in managing local disease at the primary tumor site, compared with the regional site (7.6% vs. 16%, *p* = 0.02) [39]. In terms of survival, a study of 50 patients with local disease based on clinical examination and ultrasound treated with definitive RT or conventional treatment (surgery and adjuvant RT) indicated no statistically significant difference in overall (*p* = 0.18) or disease-free survival (*p* = 0.32) between the groups [40]. However, no randomized studies have evaluated the effect of primary surgery and adjuvant RT versus definitive RT. The recommended doses are 56–60 Gy at 2 Gy/d.

Management of the primary tumor summarized:

A 1–2 cm clinical excision margin resulting in negative margins.

Adjuvant RT for primary tumors >1 cm and/or absence of low-risk characteristics (negative surgical margin, negative SLNB, no LVI and no chronic immunosuppression). Adjuvant RT offers disease control, but potential benefit should always be carefully weighed against morbidity and frailty of the patient.

Recommended dose: 50–60 Gy at 2 Gy/d, 5 F/W with 1–2 cm margins.

Definitive RT may be offered to patients who are not candidates for or refuse surgery.

Recommended dose: 56–60 Gy at 2 Gy/d, 5 F/W with 1–2 cm margins.

Prophylactic regional RT in SLNB-negative patients is not recommended.

## 6. Management of the Regional Lymph Nodes

The evidence on management of patients with nodal disease is particularly scarce, as most studies are retrospective, have too few patients and/or short follow-up periods and often have selection bias in patients receiving adjuvant therapy. Recent large single-institution studies suggest no difference between SLNB-positive patients treated with radiotherapy or therapeutic lymph node dissection (TLND) [41,42]. Patients with nodal disease have poor prognosis and high recurrence rates, compared to patients with localized disease [8,43]. This may warrant an aggressive treatment approach, despite lack of evidence on optimal management of patients with nodal disease.

### 6.1. Lymph Node Dissection and Locoregional Radiotherapy

All patients with pathologically confirmed regional lymph node metastases should be recommended TLND, whether it be a positive SLN or a palpable/radiologically confirmed metastasis. Additionally, TLND provides prognostic information as SLNB-positive patients with 1 or more positive non-SLNs are associated with a significantly worse prognosis [42]. Adjuvant regional RT is associated with increased morbidity, while the effect of adjuvant RT on OS seems less convincing, as stage III-patients (*n* = 2065) treated with surgery and adjuvant RT were not shown to have a statistically significant improvement in OS, compared to patients treated with surgery alone (*p* = 0.80) [28,36,41]. Indications for post-operative regional RT may therefore be restricted to patients with extracapsular disease to achieve regional control. The recommended doses are 50–60 Gy at 2 Gy/d, 5 F/W (Figure S3) [33]. No randomized controlled trials have compared regional nodal surgery to regional RT. Based on the above considerations and uncertainties, patients with nodal involvement should be evaluated individually in multidisciplinary tumor board consultations.

### 6.2. Definitive Radiotherapy – Nonresectable Disease

Definitive RT offers clinically meaningful disease control and is indicated for patients, who are not candidates for complete, gross resection or refuse surgical intervention. A systematic review and analysis of 23 studies found that 127 regional nodal sites treated with definitive RT resulted in a 16% recurrence rate with a median follow-up time of 24 months [39]. A prospective trial examining patients treated with macroscopic (*n* = 24) and microscopic (*n* = 26) nodal disease did not show a statistically significant difference in disease-specific survival when patients were treated with definitive RT versus TLND with/without regional RT (*p* = 0.9 and *p* = 0.7, respectively) [44]. The recommended dose to the regional lymph nodes is 56–60 Gy at 2 Gy/d per day. Collectively, definitive RT may offer clinically acceptable primary site and regional disease control for patients with unresectable disease or patients with serious co-morbidity preventing surgical intervention.

### 6.3. Other Treatment Regimens

Adjuvant chemotherapy is not recommended for resected stage III patients due to lack of association with improved OS (*n* = 2065, *p* = 0.71) [28]. Clinical trials with immunotherapy may be considered, albeit more specific recommendations will await the results of clinical studies, e.g., the ADAM trial—a randomized phase 3 trial with adjuvant avelumab in 100 stage III MCC patients [45].

Management of the regional lymph nodes summarized:

Therapeutic radical lymph node dissection is recommended in MCC patients with regional nodal involvement.

Adjuvant regional RT should be considered in the presence of extracapsular disease.

Recommended dose: 50–60 Gy at 2 Gy/d, 5 F/W.

Definitive RT should be offered to patients, who are not candidates for or refuse surgery.

Recommended dose: 56–60 Gy at 2 Gy/d, 5 F/W.

Patients with nodal involvement should be evaluated in multidisciplinary tumor board conferences.

## 7. Management of Distant Metastatic Disease

Traditionally, patients with distant metastatic disease were treated with conventional chemotherapy mainly with only palliative effect. There are currently no randomized controlled trials comparing chemotherapy with immunotherapy, however promising clinical trials have resulted in the recommendation of immunotherapy in the first-line setting in several recent guidelines [46,47].

### 7.1. Immune Checkpoint Inhibitors

Treatment with immune checkpoint inhibitors in the first-line setting is associated with response rates of >50%; some durable responses and are well tolerated [48]. Qualitative interviews with patients (*n* = 19) treated with both first-line chemotherapy and second-line treatment with the immune checkpoint inhibitor avelumab indicate a better quality of life during avelumab treatment [49]. This may be considered in the clinical setting, as MCC patients have a high average age and often multiple comorbidities.

Immunotherapy has been investigated in nonresectable stage IIIB and stage IV patients. There are no known predictive factors, as investigations of specific biomarkers (tumor PD-L1 expression, infiltrating lymphocyte PD-L1 expression, viral status, intratumoral CD8+ infiltration) have been unable to predict clinical response [2,50]. Exclusion criteria include ECOG Performance Status ≥2, steroid use in doses >10 mg prednisone daily, continued need of other immune suppressing agents, organ transplant recipients (heart, lungs and liver) or autoimmune disease with risk of unmanageable flares.

### 7.2. Avelumab (PD-L1 Antibody)

First-line setting: Treatment with avelumab in patients (*n* = 29) with metastatic MCC, no prior systemic treatment and at least 3 months-follow-up resulted in an objective response rate (ORR) of 62.1% (18/29 patients) with 4 (13.8%) complete responses (CR) and 14 (48.3%) partial responses (PR) [51]. At time of analysis, 14/18 (77.8%) of responses were ongoing with a 6-month response duration in 83% of responding patients. Among 39 patients evaluable for safety analysis, 28/39 (71.8%) experienced grade 1–3 treatment-related adverse events (TRAE) and 6/39 (15.4%) patients discontinued treatment. There were no grade 4 TRAE or treatment-related deaths.

Second-line setting: The largest clinical trial with immune therapy in stage IV MCC patients (*n* = 88) who had progressed after receiving chemotherapy resulted in an ORR of 29/88 (33%), including 19 (21.6%) PR and 10 (11.4%) CR [52]. Among the responders, the majority (74%) had duration of response of ≥1-year, while the 1-year OS rate was 52%. TRAE occurred in 62/80 (70%) of patients, including 4 (5%) patients with grade 3 TRAE. There were no deaths related to treatment [2]. These findings resulted in avelumab becoming the first approved drug for metastatic MCC by the Food and Drug Administration (FDA) and the European Medicines Agency (EMA) [53].

### 7.3. Pembrolizumab (PD-1 Antibody)

First-line setting: 50 stage IIIB/stage IV patients were administered pembrolizumab with an ORR of 56% (28/50 patients) including 12 with CR and 16 with PR [54]. Of the 28 patients with a confirmed response, the median response duration was not reached (ranged from 5.9 months to 34.5+ months) with an estimated 24 months-progressive-free survival of 48.3%. TRAE occurred in 48 (96%) of patients, including 14 (28%) patients with grade 3–4 TRAE. There was one treatment-related death. Pembrolizumab is approved for MCC in the US but not in Europe.

### 7.4. Other Immune Checkpoint Inhibitors

Case studies and clinical trials with other immune checkpoint inhibitors such as nivolumab have shown efficacy in treatment of metastatic MCC, but no applications for approval by the FDA or EMA have yet been filed [55].

### 7.5. Chemotherapy

Chemotherapy used for metastatic MCC is associated with high response rates, but responses are short-lived and the risk of adverse events, such as hematological toxicity and treatment-related death is not negligible [48]. Furthermore, the impact on OS is unclear since there are no comparisons with best supportive care. However, historical data of chemotherapy across regimens and centers over time has not indicated a benefit [28,56].

First-line setting: Response rates range from 53–61% with a median progression-free survival of 3.1 months and duration of response <8 months [48]. Ninety-five percent of patients (*n* = 62) treated may eventually develop progressive disease [57]. The primary recommended treatment regimens include cisplatin or carboplatin in combination with etoposide, as platinum-containing regimens may result in higher rates of complete (21% vs. 17%) and partial response (29% vs. 17%) compared with non-platinum-containing regimens [48,58].

Second-line-setting: Response rates range from 23–45% with a median progression-free survival of 2 months and duration of response <8 months [48].

Later-lines-setting: Chemotherapy in second-line or higher shows response rates of 10–29% with no complete responses. The median duration of response is less than 2 months with a progression-free survival and OS ranging from 2–3 months and 4–5 months, respectively [59,60].

Management of distant metastatic disease summarized:

Immune checkpoint inhibitors targeting the PD-1/PD-L1 axis should be strongly considered as first-line treatment for fit patients with no contraindications.

Avelumab has been approved by the FDA and EMA, whereas pembrolizumab has only been approved by the FDA. Small studies show effect of other checkpoint inhibitors, including nivolumab.

Immune checkpoint inhibitors are associated with high response rates, durable responses and relatively few adverse events.

Chemotherapy is primarily recommended for fit patients with contraindications to immunotherapy or after progression on immunotherapy (second line).

Recommended regimens are cisplatin or carboplatin in combination with etoposide.

No standard treatment can be recommended for patients progressing after chemotherapy; however, fit patients may be candidates for clinical trials.

## 8. Follow-Up

The patient follow-up exam should include full skin inspection and lymph node examinations with/without US of the regional lymph nodes. As 90% of MCC reoccurrences are seen within 2 years, follow-up is recommended every 3 months during this period, followed by every 6 months for the following 3 years [61]. PET-CT is the imaging modality of choice. It may be conducted one to two times a year or as clinically indicated [16,58]. Follow-up may be individualized based on patient risk factors, as there is no solid evidence for the optimal follow-up strategy in MCC patients. Risk factors, such as virus-negative and/or immune-compromised patients, may warrant closer follow-up due to increased risk of disease progression and MCC-related death [6].

Follow-up summarized:

Five-year follow-up period: Every 3 months for the first 2 years. Every 6 months for the following 3 years.

PET-CT is performed one to two times a year or if clinically indicated.

## 9. Registration

MCC is a very rare skin cancer with a dismal prognosis compared even to melanoma, and therefore a prospective systematic registration of the patients’ clinicopathological characteristics, surgical and oncological treatments and clinical outcome is paramount to improve the patient outcome. A Danish national database is under planning and all medical specialties involved in diagnosis and treatment of these patients will participate in this registration.

## 10. Methods

The current recommendations were developed by a national, multidisciplinary expert group involved in the management of Merkel cell carcinoma patients. A systematic literature review was performed using a broad search with the following key word “Merkel Cell Carcinoma” in PubMed. The literature search was performed including papers from 1998 to 2019 with exclusion of non-English papers. Additional papers were included if found in reference lists. Relevant websites with guidelines by other MCC groups and organizations such as the European Organisation for Research and Treatment of Cancer and the National Comprehensive Cancer Network were also included. Where no firm conclusions could be made based on the retrieved literature, expert consensus was obtained during discussions in the expert group. A formal evaluation of the evidence level in the retrieved references was not performed. The current work is the first attempt to agree on national guidelines, and future updates will include more formal evaluation of the literature.

## 11. Conclusions

This review constitutes the consensus management recommendations developed by the Danish MCC expert group and is based on a systematic literature search. MCC is rare, and there is a lack of randomized controlled trials. Patients with localized disease are generally recommended surgical excision with a 1–2 cm margin and adjuvant radiotherapy to the primary site. Clinically node-negative patients should be offered sentinel node biopsy. Patients with regional lymph node involvement are recommended complete node removal and, in case of extracapsular disease, adjuvant radiotherapy. Definitive radiotherapy is recommended for patients not amenable for surgery. Although available data on radiotherapy are conflicting, results from large datasets including a recent metanalysis point to more restricted dosing, ensuring sufficient margins and lack of survival benefit in patients with nodal involvement [28,30,31,32]. The latter is interpreted as a consequence of early subclinical metastatic spread. Immune checkpoint inhibitors targeting the PD-1/PD-L1 axis should be considered as first-line treatment for fit patients with nonresectable stage IIIB/IV disease.

Prospective comparative data on harmonized patient characteristics and treatment modalities are needed to determine optimal treatment sequence and modus of MCC patients with primary and regional nodal disease. Acknowledging the facts that MCC is highly immunogenic and immunotherapy is considered the new standard treatment in the advanced setting, the future role of immunotherapy in MCC patients with primary and regional disease is highly awaited [45,62].

## Figures and Tables

**Table 1:** Immunohistochemical markers in MCC and common differential diagnosis (Llombart et al. [11]).

Cancer Type	CK (AE1/AE3)	CK20	CK7	TTF-1	CD56	LCA	S-100	CD-99	Chromogranin A	Synaptophysin
Merkel cell carcinoma	+	+	−	−	+	−	−	+	+	+
Small cell lung cancer	+	−	+	+	+	−	−	+	+	+
Lymphoma	−	−	−	−	−	+	−	−	−	−
Melanoma	−/(+)	−	−	−	+	−	+	−	−	−
Ewing’s sarcoma	−/(+)	−	−	−	+	−	-	+	−/(+)	−/(+)

**Table 2:** Impact of staging with PET-CT adapted from Hawryluk et al. [16].

Stage	Patients Upstaged by PET/CT	Patients Upstaged by PET/CT (%)
IA	0/12	0%
IB	0/5	0%
IIA	0/3	0%
IIB	0/5	0%
IIIA	1/22	4.5%
IIIB	4/8	50%
IV	5/5	100%

**Table 3:** Five-year overall survival with confidence interval (CI) [8].

Stage	Clinical Staging (95% CI)	Pathological Staging (95% CI)
I	45.0% (41.9–48.1%)	62.8% (59.6–65.8%)
IIA	30.9% (27.0–34.9%)	54.6% (49.3–59.7%)
IIB	27.3% (16.0–39.9%)	34.8% (25.6–44.1%)
IIIA	Data lacking	40.3% (37.5–43.0%)
IIIB	Data lacking	26.8% (23.4–30.4%)
IV	Data lacking	13.5% (11.0–16.3%)

## Supplementary Materials

The following are available online at https://www.mdpi.com/2072-6694/12/3/554/s1, Figure S1: The 8th edition UICC staging system, Figure S2: Treatment algorithm for MCC, Figure S3: Radiotherapy indication and doses for patients with MCC.
